# Strong amplification of quantitative genetic variation under a balance between mutation and fluctuating stabilizing selection

**DOI:** 10.1093/genetics/iyag063

**Published:** 2026-03-06

**Authors:** Jason Bertram, Zahra Shafiei

**Affiliations:** Department of Mathematics, University of Western Ontario, London N6A 3K7 Canada; Department of Mathematics, University of Western Ontario, London N6A 3K7 Canada

**Keywords:** mutation–selection balance, moving-optimum models, fluctuating environments

## Abstract

The observation of high heritability in most quantitative traits has been a long-standing puzzle. There is a general consensus that simple models of quantitative genetic variation, which are mostly founded on the assumption of mutation–selection balance in a constant environment, have failed to explain high heritability. To make matters worse, the reasons for failure are unknown, leaving little to guide future model developments. Here we revisit this puzzle by taking the canonical Latter–Bulmer (LB) model and relaxing the assumption of perfect environmental stasis. Instead we assume that the trait optimum changes slowly but steadily in a stochastic manner, similar to models used for phylogenetic comparative methods. We show that our model behaves qualitatively differently to its stationary optimum counterpart even though the optimum only changes slowly. This is the result of a feedback between the adaptation rate and selection coefficient fluctuations. The heritability predictions resulting from this feedback are more consistent with observations and also less sensitive to evolutionary parameters than the classical LB model. We derive a simple formula to predict genetic variation in our model which helps to explain some of our counter-intuitive results and which should be useful for understanding the potential influence of fluctuations in future work. Since the feedback driving our results should also occur in more complex models e.g. with multiple traits, we argue that environmental change has been an essential biological ingredient missing in most previous mutation-selection balance models of quantitative trait heritability.

## Introduction

Quantitative traits exhibit abundant genetic variation even when under strong selection, with narrow-sense heritability typically being in the range $h^{2}=0.1-0.6$ (Mousseau and Roff 1987; Johnson and Barton 2005). This is surprising because selection usually reduces genetic variation. We therefore expect to see abundant variation or strong selection, not both (Walsh and Blows 2009).

High variation despite strong selection can be explained by mutations if the influx of new variation is sufficiently large—the mutation-selection balance (MSB) hypothesis. To explore and test the MSB hypothesis, quantitative genetic models have been developed to predict variation under MSB. To date these models have failed to convincingly reconcile high heritability and strong selection because the only way to have both is if the trait’s mutational target is huge (Turelli 1984; Johnson and Barton 2005; Huang et al. 2016; Walsh and Lynch 2018; Sella and Barton 2019).

Multiple lines of evidence support high polygenicity in many traits, including quantitative trait loci experiments (Hill and Kirkpatrick 2010), evolve and resequence experiments (Barghi et al. 2019; Kelly and Hughes 2019) and genome-wide association studies (Sella and Barton 2019). However, the demands of MSB models are severe. The canonical MSB models are the Latter–Bulmer (LB) (Latter 1960; Bulmer 1972), and “house of cards” models (Turelli 1984). Both models assume a single trait under direct stabilizing selection with a static optimum, and make identical heritability predictions. In *D. melanogaster*, these models require ∼1% of the entire euchromatic genome to contribute to each trait at minimum to reach observed heritabilities (Huang et al. 2016) (also see Required mutational target sizes). Moreover, predicted heritability is sensitive to the model parameters (mutation rate, intensity of selection and number of loci) (Bürger et al. 1989). Therefore, to be consistent with observations, canonical MSB models require tight constraints on evolutionary parameters including consistently large mutational target sizes (Bürger et al. 1989; Sella and Barton 2019).

Canonical MSB models also fail to account for pleiotropy, which must be prevalent if mutational targets are large. The importance of pleiotropy can also be seen at the phenotypic level, because only a modest number of traits can be independently under strong selection without imposing an unbearable cost to fitness (Johnson and Barton 2005). In the presence of pleiotropy a key unresolved issue is the relationship between the effect of alleles on a given trait and the corresponding effect on fitness (Sella and Barton 2019).

Here we do not address the formidable challenges related to pleiotropy, but instead focus on a another unrealistic assumption of canonical MSB models: perfect environmental stasis. We show that the single-trait LB model is much better at predicting realistic heritabilities when this assumption is relaxed. Environmental fluctuations have long been recognized as an important determinant of genetic diversity (Kimura 1954; Bell 2010), and are ubiquitous in nature (Kingsolver and Diamond 2011). There is a sizeable literature on environmental fluctuations in population genetics, but with an overwhelming focus on fluctuations as a source of balancing selection (Hedrick 2006; Yi and Dean 2013; Wittmann et al. 2017; Bertram and Masel 2019; Yamamichi et al. 2023). Our understanding of mutation-selection-balance in fluctuating environments is rudimentary in comparison.

In single-locus haploid models, simply introducing selection coefficient fluctuations does not by itself create a balancing effect (Dempster 1955; Haldane and Jayakar 1963). Selection coefficient fluctuations also do not amplify heterozygosity in mutation-selection balance in many studies (Takahata and Kimura 1979; Huerta-Sanchez et al. 2008). We will show that there is a close connection between single-locus models and the multi-locus quantitative trait model of interest here. However, in our results heterozygosity is amplified by fluctuating selection. This occurs not because the quantitative trait model introduces a balancing effect, but rather because fluctuations counteract the tendency of stabilizing selection on a trait to eliminate new mutations. Single locus models miss this effect if they assume that new mutations are neutral on average (Takahata and Kimura 1979; Huerta-Sanchez et al. 2008).

In (multi-locus) quantitative genetic models, environmental change is typically modeled as movement of the optimal phenotype (Kopp and Matuszewski 2014). Optimum movement makes it difficult to predict the additive genetic variance $V_{g}$—even at steady state—and as a result $V_{g}$ has frequently been treated as a model input. This has particularly been the case in moving optimum models of extinction and evolutionary rescue, which are typically interested in shorter timescales where it makes sense to approximate $V_{g}$ as constant (Bürger and Lynch 1995; Lande and Shannon 1996; Osmond and Klausmeier 2017; Klausmeier et al. 2020; Chevin and Bridle 2025). Newer approaches explicitly model the dynamics of the phenotype distribution (Garnier et al. 2023), but currently rely on simplifying genetic assumptions such as the infinitesimal model (Barton et al. 2017). Consequently, this class of models does not directly address the heritability question of interest here, which requires predicting genetic variance without presupposing many contributing loci of small effect.

Other moving optimum models have focused on the genetic changes underpinning adaptation to a new optimal phenotype. Many of these models also do not predict genetic variance, focusing instead on other genetic details like fixation timings/probabilities and the role of large allele frequency changes at a few loci (sweeps) versus small allele changes at many loci (polygenic shifts) (Kopp and Hermisson 2007, 2009a, 2009b; Jain and Stephan 2015; Matuszewski et al. 2015; Jain and Stephan 2017a).

Among the studies that have predicted quantitative genetic variation in changing environments, many have studied the case of a single, sudden environmental shift (Barton and Turelli 1987; Jain and Stephan 2017b; Thornton 2019; Hayward and Sella 2022). Large increases in genetic variation can occur as alleles needed to match the new optimum rise in frequency, albeit only transiently before a new stationary-optimum equilibrium is reached.

If optimum movement is sustained, e.g. cyclically (Kondrashov and Yampolsky 1996; Bürger and Gimelfarb 2002) or at a constant velocity (Bürger 1999; Waxman and Peck 1999; Bello and Waxman 2006), genetic variation can remain substantially elevated compared to the stationary case. Similar to the case of a sudden shift, this effect is due to allele frequency increases driven by displacement of the trait optimum, not balancing selection. However, the amount of genetic variation is sensitive to the assumed model of environmental change e.g. the amplitude and frequency of environmental cycles (Bürger and Gimelfarb 2002) or enforcing unidirectional optimum movement (Bürger 1999; Waxman and Peck 1999). Thus, while it is known that environmental change can greatly increase $h^{2}$, the issue is that special types of change have thus far been required to obtain this result, leaving it unclear how applicable these results are to observations (Walsh and Lynch 2018).

Here we assume that the trait optimum undergoes Brownian motion. Brownian motion and the closely related Ornstein-Uhlenbeck (OU) process (Brownian motion with a “restoring force” preventing unbounded movement) is widely used in phylogenetic comparative methods (Hansen 1997). These models are based on the idea that fitness depends on many different environmental factors which are continually changing, causing the optimal trait value to wander through trait space in unpredictable ways. This slowly accumulating undirected stochastic movement is presumably commonplace in nature, unlike the more situational properties of directional/cyclical/pulse moving optimum models. We focus on Brownian motion rather than the more general OU because OU has equivalent behavior unless the restoring force is strong (discussed further in Fluctuations in optimum). Moving optimum quantitative genetic models with similar assumptions have been studied previously, but did not predict genetic variance (Lande and Shannon 1996; see above).

Apart from introducing environmental fluctuations we follow classical LB assumptions (Latter 1960; Bulmer 1972). Namely, we assume a single additive trait under Gaussian stabilizing selection, perfect linkage equilibrium and biallelic loci, although unlike LB we do account for genetic drift (Bürger et al. 1989).

Our model, henceforth the “fluctuating optimum model”, behaves qualitatively differently to its stationary optimum counterpart even though the optimum only changes slowly. As the optimum changes, allele frequencies adjust to chase the optimum, and hence so does the additive genetic variance $V_{g}$. But $V_{g}$ also controls the rate at which the population chases the optimum. Thus, we have a feedback: $V_{g}$ influences the time-course of selection, which in turn influences $V_{g}$. Using a combination of simulations and mathematical analysis, we show that the heritability predictions resulting from this feedback are more consistent with observations and also less sensitive to evolutionary parameters than the classical LB predictions.

## Methods

### Stabilizing selection in a constant environment

We first briefly review the LB model (Latter 1960; Bulmer 1972). A quantitative trait with value denoted *z* is determined by additive contributions from *L* bi-allelic diploid loci, where individuals with trait value *z* have relative fitness


(1) $$W(z)=e^{-\frac{(z-z^{*}{)}^{2}}{2V_{s}}}.$$

Here $z^{*}$ is the trait optimum and the selection strength parameter $V_{s}$ is assumed to be much larger than the total trait variance in the population.

Assuming linkage equilibrium between loci, the allele frequency change due to selection obeys the differential equation (Wright 1935; Barton 1986)


(2) $$\frac{dp_{i}}{dt}=\left[\frac{a_{i}^{2}}{2V_{s}}(2p_{i}-1)+\frac{a_{i}}{V_{s}}\delta \right]p_{i}(1-p_{i}),$$

where $p_{i}$ is the frequency of the focal allele at locus *i*, $\delta =z^{*}-\bar{z}$ is the displacement of the trait mean $\bar{z}$ from the optimum, and $a_{i}$ is the effect on *z* of switching one copy of a gene from the non-focal to the focal allele. Note that while Equation (2) is conventionally derived assuming a normal trait distribution (Barton 1986; Walsh and Lynch 2018), it also holds without normality (Jain and Stephan 2017b). This is important because in some parameter regimes the number of segregating loci in our model is too small to assume normality.

The LB model assumes the population is at the optimum (δ=0). The remaining term in Equation (2) is negative for $p<\frac{1}{2}$ and positive for $p>\frac{1}{2}$ implying disruptive selection on allele frequency. Henceforth we will call this component of stabilizing selection “disruptive selection” to distinguish it from the directional selection driven by the optimum displacement *δ*.

The LB model thus has a selection coefficient


(3) $$s=\frac{a_{i}^{2}}{2V_{s}}$$

acting to purge low frequency alleles, including new mutations. This is counteracted by a mutation influx of *μ* per allele copy per locus per generation. We assume forward and backward mutation rates are equal, which implies that the expected rate of change in $p_{i}$ due to mutation is $\mu (1-2p_{i})$. In mutation-selection balance, minor alleles segregate at frequency $p_{i}\approx \mu /s$, and so the total additive genetic variance is given by (Latter 1960; Bulmer 1972)


(4) $$V_{g}^{LB}=2\sum_{i}a_{i}^{2}p_{i}(1-p_{i})\approx 4L\mu V_{s}.$$

This is the same prediction as the house of cards model (Turelli 1984; Johnson and Barton 2005). Since the LB model assumes no genetic drift, Equation (4) breaks down in a finite population, but is approximately true provided that selection is strong enough to overwhelm drift at the mutation-selection balance frequency $p_{i}∼\mu /s$ (Bürger et al. 1989).

Equation (4) has the nice property of being independent of the effect sizes $a_{i}$. In the model developed below, things are not so simple. To improve tractability we assume that effect size magnitudes are identical at all loci $|a_{i}|=a$. This assumption can be problematic if the optimum is stationary because it produces modeling artefacts where $\bar{z}$ cannot reach $z^{*}$ since each individual’s *z* is artificially constrained to be a multiple of *a* (Barton 1986). In the fluctuating optimum model this is less of a concern because $z^{*}$ is continually moving.

### Fluctuations in optimum

We now allow the optimum to move by assuming $z^{*}=z_{t}^{*}$ follows a Brownian motion process with variance $\sigma^{2}$ per generation. We use a Brownian model even though it permits unbounded movement of $z_{t}^{*}$ because the more complicated OU model generates essentially the same optimum displacements $\delta_{t}$ in many cases. By using a Brownian model we are assuming that the population adapts so quickly to optimum changes that the restoring force on $z_{t}^{*}$ has no noticeable effect on $\delta_{t}$. This assumption breaks down if the restoring force is strong enough to constrain the long-term variance in $z_{t}^{*}$ to be comparable to the environmental trait variance $V_{e}$ (Ornstein-Uhlenbeck process for $\delta_{t}$).

As $z_{t}^{*}$ moves and $\delta_{t}$ grows, allele frequencies start to shift according to Equation (2). These shifts drive $\bar{z}$ towards $z_{t}^{*}$ (Thornton 2019; Hayward and Sella 2022). The rate of pursuit is approximately (Approximate constancy of $V_{g}$; Lande 1976; Barton and Turelli 1987; Hayward and Sella 2022)


(5) $$\frac{d\bar{z}}{dt}\approx v\delta_{t}.$$

where $v=V_{g}/V_{s}$. Consequently, $\delta_{t}=z_{t}^{*}-\bar{z}$ approximately obeys the Itô stochastic differential equation (SDE)


(6) $$d\delta_{t}=-v\delta_{t}\;dt+\sigma \;dW_{t}$$

where $W_{t}$ is a Brownian motion process with unit variance.

In the following analysis, $\delta_{t}$ remains small enough that $V_{g}$ can be treated as approximately constant once the population reaches mutation-selection balance (Approximate constancy of $V_{g}$). Equation (6) then describes an OU process with zero mean, variance $\sigma^{2}$ and restoring force *v*.

The key property of the OU process Equation (6) used in the analysis below (Diffusion approximation) is the steady-state temporal autocovariance of $\delta_{t}$, given by the standard formula


(7) $$\langle \delta_{t},\delta_{t-\tau }\rangle =\frac{\sigma^{2}}{2v}e^{-v\tau }.$$

The autocovariance thus decays exponentially with timescale $\tau_{a}\equiv v^{-1}$.

### Simulation

We simulate the dynamics of $p_{i}$ at each locus *i* in discrete time using a discretized version of Equation (2) to incorporate selection, and Wright-Fisher sampling to incorporate genetic drift. That is, the abundance of the focal allele at locus *i* in generation t+1 is sampled from a binomial distribution with 2N trials and success probability $p_{i}(t)+\frac{dp_{i}}{\;dt}\Delta t$ where the timestep Δt=1 represents one generation. This procedure implicitly enforces perfect linkage equilibrium since the $p_{i}$ evolve independently at each locus apart from the selective coupling through *δ*.

Mutations are generated as a Poisson process at rate *μ* per locus per allele copy, with equal back/forward rates between the two possible alleles. When a mutation creates a new allele at locus *i*, it is assigned a trait effect $a_{i}=\pm a$ with with equal +/− probability. Brownian movement of the optimum is approximated in discrete time as $z_{t+1}^{*}=z_{t}^{*}+\epsilon$ where ϵ is a normal random variable with mean 0 and variance $\sigma^{2}$. To reach mutation-selection-drift balance, each replicate population is simulated for 20N generations. Genetic variance is computed as $V_{g}=2a^{2}\sum_{i=1}^{L}p_{i}(1-p_{i})$.

### Parameters

Our parameter choices are summarized in Table 1, with some explanation provided below. We choose our trait units such that $V_{e}=1$, which means $V_{g},V_{s},a^{2}$ and $\sigma^{2}$ are all measured relative to $V_{e}$. In particular, the narrow-sense heritability becomes


(8) $$h^{2}=\frac{V_{g}}{V_{g}+1}$$

In addition to the canonical $V_{s}=20$ (Turelli 1984) we also include a stronger selection $V_{s}=5$ scenario following (Kingsolver et al. 2001; Johnson and Barton 2005). Following Sella and Barton (2019), we assume an upper bound of $a^{2}\leq 0.1$ on allele effect sizes to remain in the quantitative trait regime where individual alleles do not have large phenotypic effects.

**Table 1.** Summary of parameters and their assumed values.

Parameter	Symbol	Value(s)
Selection strength	$V_{s}$	5,20
Mutation rate	*μ*	$10^{-7}-10^{-5}$
# of contributing loci	*L*	$10-10^{3}$
Effective population size	2N	$10^{2}-10^{5}$
Mutation effect size	$a^{2}$	$10^{-3}-10^{-1}$
Fluctuation intensity	$\sigma^{2}$	$0-10^{-2}$

In most of our results we assume $2N=10^{4}$ for the drift-effective population size. This ensures 2Ns>1 even if $a^{2}=10^{-2}$, so that disruptive selection is not swamped by genetic drift. Other *N* values are included to explore the role of drift.

We primarily use the mutation rate $\mu =6.6\times 10^{-6}$/kb from a recent *D. melanogaster* mutation accumulation experiment (Huang et al. 2016), although results for other mutation rates are included for reference. We assume each locus is a kilobase block, roughly corresponding to a gene, rather than modeling mutations at individual base pairs.

With the above parameter values, we need $L=10^{3}$ ($=10^{6}$ bp) to reach $V_{g}^{LB}=0.1$ (the lower end of the empirical range) in the LB model under strong selection ($V_{s}=5$). Since $L\geq 10^{3}$ implies an enormous genomic footprint for each trait (Huang et al. 2016), and we wish to demonstrate that it is not necessary to assume such large *L* values, we assume $L\leq 10^{3}$. The mutational heritability (influx of heritability due to mutation) is then at most $4L\mu a^{2}=2\times 10^{-3}$. This is on the higher end of empirical estimates but not implausibly large (Johnson and Barton 2005). We assume slow environmental change $\sigma^{2}\leq 10^{-2}$.

## Results

### Simulation results: fluctuations greatly increase heritability

**Fig. 1.**
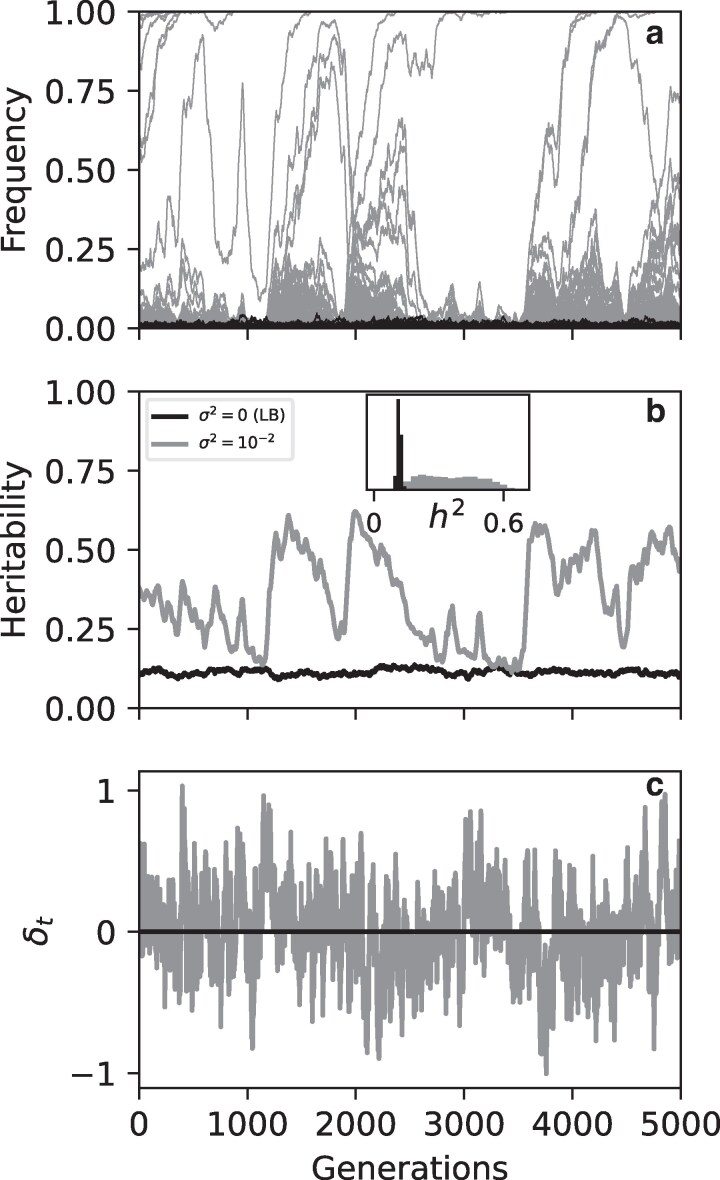
Example simulation of the fluctuating optimum model ($\sigma^{2}=10^{-2}$; grey) and constant optimum model ($\sigma^{2}=0$; black) showing the strong increase of genetic variation in the presence of fluctuations. a) Allele frequencies at all L=1000 loci. Alleles cluster near p=0 because new mutations are chosen as focal allele. Loci with p=0,1 are not visible. b) Heritability is amplified but highly variable over time. Inset: histogram of heritability over time. c) Optimum displacement $\delta_{t}$ over time. In the LB case $\delta_{t}=0$. Other parameters: $2N=10^{4},a^{2}=10^{-1},V_{s}=5,\mu =6.6\times 10^{-6}$.

Fluctuations dramatically change the heritability predictions of simple quantitative genetic variation models. Fig. 1 shows an example of the dynamics involved. When the trait optimum is stationary ($\sigma^{2}=0$), new mutations are deleterious, constraining them to low frequencies. With a fluctuating optimum ($\sigma^{2}>0$), selection coefficients of new mutations are sometimes positive depending on $\delta_{t}$, releasing mutations to higher frequencies and even enabling fixation. As a result, $V_{g}$ is substantially elevated in the presence of fluctuations. In some models fluctuating selection can have an even greater impact on diversity by inducing a form of balancing selection (Yamamichi et al. 2023)—in Mechanism generating diversity we show that this is not the case in the fluctuating optimum model. Fluctuations also induce much stronger variability in $V_{g}$ over time, although $h^{2}$ is almost never smaller than the $\sigma^{2}=0$ case.

This behavior occurs without requiring rapid environmental change ($\sigma^{2}=0.01$) or particularly large optimum displacements. The standard deviation of $\delta_{t}$ is ≈0.3, implying a typical directional selection coefficient of $a|\delta |/V_{s}\approx 0.02$. At most $\left|\delta_{t}\right|$ occasionally reaches ∼1 giving $a|\delta |/V_{s}\approx 0.06$. These numbers assume effect sizes at our upper limit of $a^{2}=0.1$ and strong stabilizing selection $V_{s}=5$ (Parameters). By comparison, under the same assumptions, the corresponding disruptive selection coefficient is $s=a^{2}/2V_{s}=0.01$, a number regarded as plausible based on empirical estimates of $V_{s}$ (Johnson and Barton 2005).

Next we evaluate the parameter dependence of $h^{2}$ in the presence of fluctuations. Given the variability over time evident in Fig. 1, we simulate $10^{3}$ replicate populations and show distributions of $h^{2}$ across replicates in the final timestep. This procedure ensures independence of each simulated $h^{2}$ value. The distribution among replicates is found to be identical to the distribution over time within a simulation, implying that our among-replicate distributions also describe the population steady-state.

**Fig. 2.**
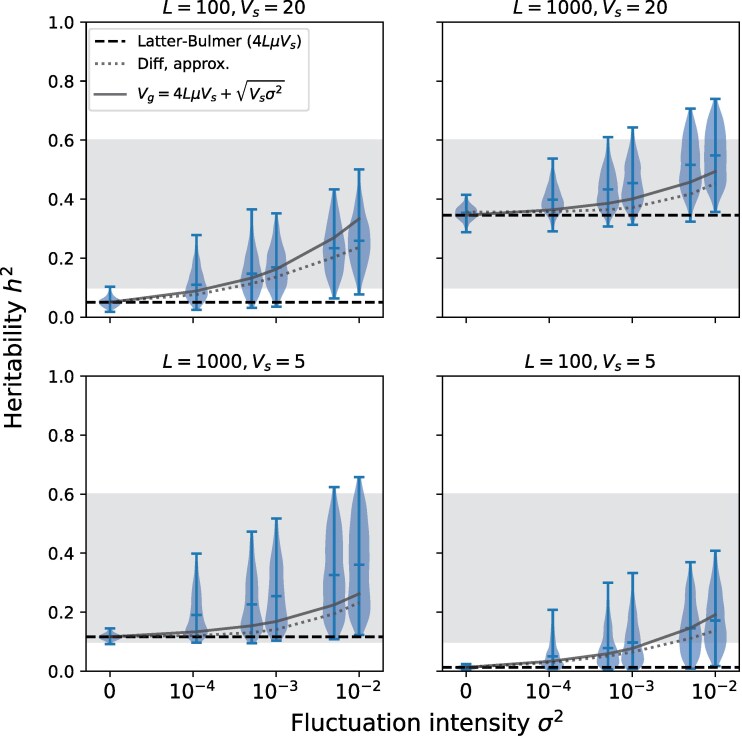
Simulated heritability in the fluctuating optimum model. For each parameter combination, $10^{3}$ replicate simulations are run and $h^{2}$ computed from $V_{g}$ in the final generation. Violinplots show the resulting distribution of $h^{2}$ among replicates with the mean marked in the center. Dashed line shows the LB prediction. Empirical range $0.1\leq h^{2}\leq 0.6$ is shaded. Dotted line: numerical solution of the diffusion approximation Equation (18). Solid line: analytical approximation Equation (19). Other parameters: $2N=10^{4}$, $a^{2}=10^{-1},\mu =6.6\times 10^{-6}$.

To evaluate how strong fluctuations need to be to meaningfully amplify $h^{2}$, we first show $h^{2}$ as a function of $\sigma^{2}$ for different combinations of *L* and $V_{s}$ (Fig. 2). Even for fluctuations as small as $\sigma^{2}=10^{-3}$, the increase in the predicted average heritability is substantial. The effect grows as $\sigma^{2}$ increases, pushing $h^{2}$ comfortably above 0.1 even for the challenging case of $V_{s}=5$ and L=100 where $V_{g}^{LB}\approx 0.01$. The increase in $h^{2}$ is noticeably smaller for $\sigma^{2}=10^{-4}$, implying $\sigma^{2}∼10^{-3}$ is needed for fluctuations to have a meaningful impact on $h^{2}$ in our simulations.

Similar to Fig. 1, fluctuations introduce substantial variance in Fig. 2. We quantify this effect explicitly in Table 2, finding coefficients of variation as high as 0.67. As a result, $h^{2}$ is frequently much higher than the replicate mean, resulting in $h^{2}$ values well above 0.1. This variance also implies $h^{2}$ values frequently smaller than the mean, though it can be seen that values as low as the LB case are rare. This also implies high variability in $h^{2}$ over time, an effect that has been documented before in MSB models (Bürger and Gimelfarb 2002).

**Table 2.** Variance and coefficient of variation (CV) of $h^{2}$.

$V_{s}$	*L*	Mean	Variance	CV
5	100	0.097	$4.3\times 10^{-3}$	0.67
20	100	0.17	$4.1\times 10^{-3}$	0.38
5	1000	0.25	$9.4\times 10^{-2}$	0.38
20	1000	0.45	$4.4\times 10^{-3}$	0.15

Values are computed from among-replicate distributions, but are identical to values obtained from the steady-state distribution over time. Parameters: $\sigma^{2}=10^{-3},2N=10^{4},\mu =6.6\times 10^{-6},a^{2}=0.1$.

**Fig. 3.**
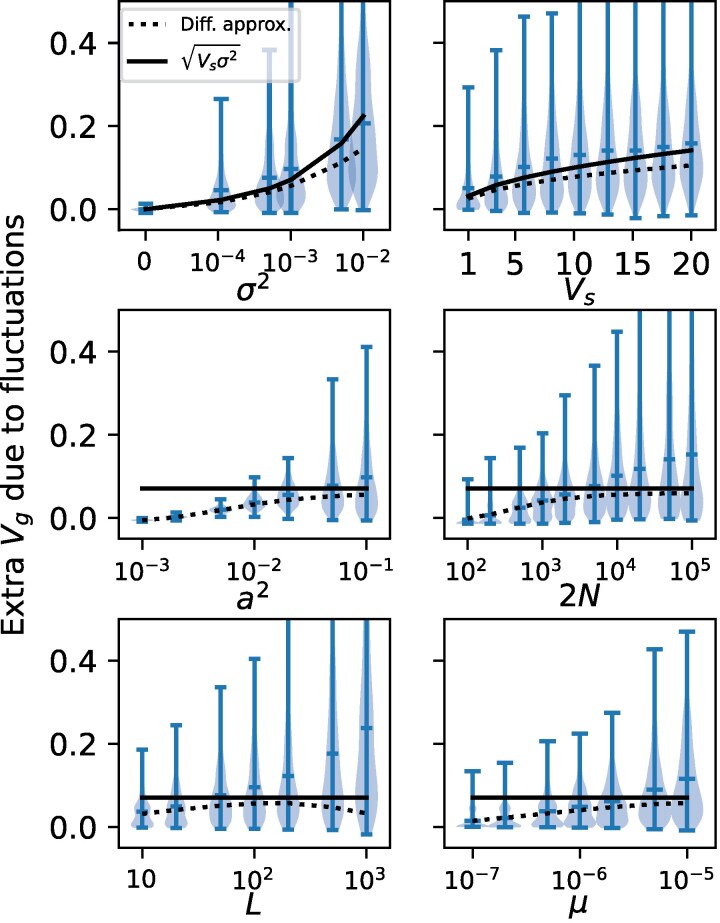
Dependence of the extra variance created by fluctuations ($V_{g}-Vg^{LB}$) on the six model parameters (Table 1). The default parameters are $\sigma^{2}=10^{-3},V_{s}=5,a^{2}=0.1,2N=10^{4},L=100,\mu =6.6\times 10^{-6}$, with one parameter allowed to vary in each respective panel. The diffusion approximation Equation (18) underestimates the extra variance for larger values of *L* and *μ* (higher mutational influx) and *N* (weaker drift), and consequently so does its approximate analytical solution $V_{g}-Vg^{LB}=\sqrt{V_{s}\sigma^{2}}$.

The genetic variance added by fluctuations $V_{g}-V_{g}^{LB}$ increases with all model parameters (Fig. 3), although unlike the LB model, there is much less sensitivity to changes in *L*, *μ* and $V_{s}$. When drift is strong (small *N*) or the effect size *a* is small the extra $V_{g}$ vanishes. Hence, strong selection on individual alleles relative to drift (2Ns≫1) is required for fluctuating selection to amplify $V_{g}$.

### Feedback between fluctuations and adaptation rate

We now turn to the analysis of the processes driving amplification of $V_{g}$ in Figs. 1–3. Fluctuations in the present model are essentially continual changes in $\delta_{t}$, yet the model’s behavior depends crucially on the extent to which $\delta_{t}$ is temporally consistent. The key quantity is the temporal autocovariance Equation (7).

To illustrate the importance of $\delta_{t}$ autocovariance, suppose $\delta_{t}$ had no autocovariance. This would require replacing Equation (6) such that $\delta_{t}$ followed a white noise process with the same variance $\sigma^{2}$ per unit time (this alternative fluctuation model is introduced for illustration only, it will not be used further). Now, assume p≪1 (due to disruptive selection) and consider a time increment Δt short enough that the p(1−p)≈p factor in Equation (2) is approximately constant. Then, from Equation (2), the accumulated allele frequency change Δp due to $\delta_{t}$ follows a Brownian motion process with variance $(ap/V_{s}{)}^{2}\sigma^{2}$ per generation. In particular, after Δt=1/s generations, we have $|\Delta p|∼\sqrt{(ap/V_{s}{)}^{2}\sigma^{2}\Delta t}=p\sigma /\sqrt{V_{s}}$. Thus we have |Δp|≪p in our parameter regime because $\sigma^{2}/V_{s}\leq 2\times 10^{-3}$ (Parameters). Over this same time Δt=1/s, disruptive selection induces a much larger change of order |Δp|∼p. Thus, without autocovariance, fluctuations would barely perturb the LB mutation-selection balance.

The effect of autocovariance in $\delta_{t}$ is harder to analyze because Equation (7) depends on $V_{g}$, which is what we want to predict. We cannot avoid this $V_{g}$ dependence because it strongly affects the autocovariance. This is apparent from the ratio of autocorrelation timescale $\tau_{a}=V_{s}/V_{g}$ to disruptive selection 1/s timescale


(9) $$\frac{V_{s}/V_{g}}{1/s}=\frac{a^{2}}{2V_{g}}$$

If $V_{g}=1$ then the above ratio is ≪1 implying autocovariance decays rapidly. If $V_{g}=10^{-2}$ then Equation (9) evaluates to ≈5, implying little autocovariance decay over the disruptive selection timescale.

Thus we have a feedback. $V_{g}$ controls the rate of adaptation, which influences $\delta_{t}$ and hence the fluctuations in selection coefficients, which in turn influences allele frequency dynamics and $V_{g}$. This feedback is what drives the heritability predictions of the fluctuating optimum model.

### Diffusion approximation

We now summarize our approach for analyzing the steady state in the fluctuating optimum model and obtain a formula for $V_{g}$. We proceed in two steps. First, we ignore the $V_{g}$ feedback and compute the distribution of allele frequencies for fixed $V_{g}$. Second, we use a self-consistency condition to resolve the feedback and solve for $V_{g}$.

In the first step, we average our model over the autocovariance timescale $\tau_{a}=V_{s}/V_{g}$. Since $\delta_{t}$ is approximately independent of $\delta_{t+\tau_{a}}$, this averaging yields an uncorrelated diffusion approximation that captures the effects of autocovariance (Steady state density; Takahata et al. 1975; Van Kampen 1992). The resulting diffusion approximation is summarized by the mean rate of change in $p_{i}$


(10) $$\left(\frac{\beta }{2}-\gamma \right)(1-2p_{i})p_{i}(1-p_{i})$$

and the variance


(11) $$p_{i}(1-p_{i})[1+\beta p_{i}(1-p_{i})]$$

where γ=2Ns, $\beta =2N\sigma_{a}^{2}$ and


(12) $$\sigma_{a}^{2}=\frac{a^{2}\sigma^{2}}{V_{g}^{2}}.$$

is the net variance in selection coefficients caused by fluctuations in the optimum. Formally, $\sigma_{a}^{2}$ adds up the autocovariance in $\frac{a}{V_{s}}\delta_{t}$ (from Equation (2)) over the autocorrelation timescale $\tau_{a}$.

The above diffusion is similar to single-locus fluctuating selection models (Kimura 1954; Karlin and Levikson 1974; Takahata et al. 1975; Huerta-Sanchez et al. 2008). Superficially the only difference is that the $\left(\beta /2-\gamma )(1-2p_{i}\right)$ factor in Equation (10) is replaced in single-locus models by β(1−2p)/2−γ, where *γ* and *β* are the corresponding single-locus parameters for the mean and variance of the selection coefficient.

The deeper difference is that *β* is not a parameter in the fluctuating optimum model. Since we have conditioned on $V_{g}$, it appears as though we have effectively reduced the multi-locus fluctuating optimum model to a single-locus diffusion in Equations (10)–(11). However, when we solve for $V_{g}$ below, *β* must be treated as a function of $V_{g}$ and the multi-locus aspect of the problem becomes apparent. The close connection with single-locus models is nevertheless instructive and will be discussed further below (Mechanism generating diversity).

From Equations (10)–(11), we calculate the distribution $\bar{\phi }(p\;|\;V_{g})$ of allele frequencies for given $V_{g}$ at mutation-selection balance (Appendix Steady state density)


(13) $$\bar{\phi }(p\;|\;V_{g})=C\frac{{\left[\;p(1-p)\right]}^{\theta -1}}{{\left[1+\beta p(1-p)\right]}^{\frac{2\gamma }{\beta }+\theta }}$$

where θ=4Nμ and $C=1/\int_{0}^{1}\bar{\phi }(p)\;dp$ is a normalization constant.

In the limit $\sigma^{2}\to 0$ we retrieve the stationary optimum distribution (Bürger et al. 1989)


(14) $$\lim_{\sigma^{2}\to 0}\bar{\phi }(p\;|\;V_{g})=C{\left[\;p(1-p)\right]}^{\theta -1}e^{-2\gamma p(1-p)}.$$

Compared with the neutral distribution $C[p(1-p){]}^{\theta -1}$, Equation (14) introduces an exponential decay term that displaces frequencies away from intermediate values. Hence $V_{g}$ is much smaller than the neutral case when disruptive selection is strong enough to overcome drift (γ>1).

With fluctuations, $\bar{\phi }$ is also depressed at intermediate frequencies compared with the neutral distribution, but not to the same extent as the stationary optimum case (Equation (14)). The factor $1/[1+\beta p(1-p){]}^{\frac{2\gamma }{\beta }+\theta }$ in Equation (13) decays slower than exponential, approaching exponential only in the limit β→0. This gives the first clue about the intensity of fluctuations required to start affecting $h^{2}$, namely that γ/β should not be very large, or in other words


(15) β∼γ,

This condition will be discussed further below.

### Mechanism generating diversity

The diffusion approximation Equaitons (10)-(11) shows that fluctuations have two effects in the fluctuating optimum model: perturbing the mean and amplifying the variance.

Considering the mean Equation (10) first, the β/2 term counteracts disruptive selection on allele frequencies. This term was mistakenly omitted in early fluctuating selection models (Kimura 1954). It arises from the nonlinearity of the selection response and is not particular to stabilizing selection. To better understand its origins, it is instructive to consider the single-locus, haploid Karlin-Levikson (KL) model (Karlin and Levikson 1974). Two alleles at a locus have fitness $1+\alpha_{1}$ and $1+\alpha_{2}$ respectively where $\alpha_{1}$ and $\alpha_{2}$ are independent and identically distributed random variables with zero mean and variance $\sigma_{KL}^{2}/2$. The expected allele frequency change over one generation is then given by


(16) $$\begin{array}{rl} E[\Delta p] & =E\left[\frac{\alpha_{1}-\alpha_{2}}{1+\alpha_{1}p+\alpha_{2}(1-p)}\right]p(1-p)\\ & \approx \frac{\sigma_{KL}^{2}}{2}(1-2p)p(1-p) \end{array}$$

Here the second line is obtained by Taylor expanding in $\alpha_{1}$ and $\alpha_{2}$ assuming $\sigma_{KL}^{2}\ll 1$ (Huerta-Sanchez et al. 2008). Hence, even though the fluctuations in fitness are symmetrical in the KL model, E[Δp] has negative frequency dependence centered at $p=\frac{1}{2}$. A similar negative frequency dependence occurs in Equation (10) when β/2>γ.

This suggests that fluctuations might induce stable polymorphism in the KL and fluctuating optimum models (see Yamamichi et al. 2023 for a recent review of this phenomenon). However, polymorphism is in fact not stable due to the other effect of fluctuations: amplifying the diffusive term Equation (11).

In the KL model, the additional dispersion due to fluctuations exactly negates the negative frequency dependence in Equation (16). As a result, there is no stabilizing effect (Huerta-Sanchez et al. 2008). This makes sense because the source of negative frequency dependence in Equation (16) is division by the mean fitness (denominator of the first line). The classical analysis of fluctuating selection in haploid single-locus models (Dempster 1955; Haldane and Jayakar 1963) is based on the dynamics of the ratio p/(1−p) which is independent of mean fitness—that analysis also concludes that fluctuation does not stabilize polymorphism in simple haploid models like the KL model.

For similar reasons, there is no stabilizing effect of fluctuations in the fluctuating optimum model. This can be seen from the steady state distribution $\bar{\phi }$ in Equation (13). If polymorphism was stable then, in the absence of mutation and genetic drift (i.e. considering the action of selection alone), $\bar{\phi }$ would be a probability density with nonzero probability for p>0 (Dean and Shnerb 2020). Setting μ=0 and letting N→∞ to remove mutation and drift respectively we obtain


(17) $$\bar{\phi }\propto [p(1-p){]}^{-1-\frac{2\gamma }{\beta }},$$

Since *γ* and *β* are positive, the area under this function diverges to infinity near p=0 and p=1. Hence it is not a probability density, proving the absence of stable polymorphism (Dean and Shnerb 2020). This conclusion holds regardless of the sign of β/2−γ i.e. independently of any negative frequency dependence in Equation (10).

The reason that fluctuations generate genetic diversity in the fluctuating optimum model is that they weaken the influence of disruptive selection allowing alleles to segregate at higher frequencies. Analyzing allele frequency displacements under the diffusion Equations (10)–(11), shows that β∼γ (Equation (15)) is required for fluctuations to start overpowering disruptive selection and release alleles to higher frequencies (Balance of forces on allele frequencies).

There is no inflation of variance in the absence of disruptive selection. In fact, fluctuations decrease variation relative to the neutral case $\bar{\phi }\propto [p(1-p){]}^{\theta -1}$ if we set γ=0 in Equation (13). Moreover, note that although the condition β∼γ approximately coincides with where the mean change in Equation (10) vanishes (β/2=γ), disruptive selection is not nullified in this case and variation is still depressed relative to the neutral case. It is only in the strong fluctuation limit β→∞ that Equation (13) approaches the neutral distribution with all hint of disruptive selection purged. This neutral case thus represents the greatest diversity that fluctuations can achieve, consistent with the absence of a stabilizing effect.

### Solving for the steady state $V_{g}$

We now proceed to the second step of the diffusion analysis: finding the steady-state $V_{g}$ by applying a self-consistency condition to $\bar{\phi }(p\;|\;V_{g})$. First, we assume allele frequency dynamics are independent at each locus. Then $\bar{\phi }(p\;|\;V_{g})$ gives the steady-state distribution of allele frequencies among loci for a given $V_{g}$. Therefore, for consistency the genetic variation implied by $\bar{\phi }$ should equal $V_{g}$:


(18) $$V_{g}=2La^{2}\int_{0}^{1}p(1-p)\bar{\phi }(p\;|\;V_{g})\;dp$$

Our diffusion approximation for the steady-state $V_{g}$ (dotted lines in Figs. 2 and 3) is the solution of Equation (18). This solution is obtained by numerically integrating Equation (18).

The diffusion approximation Equation (18) does a good job capturing the parameter dependence of the added $V_{g}$ (dotted lines in Fig. 3) with the exception of the higher values of *μ*, *L* and *N* where the diffusion approximation is a substantial underestimate. This is due the breakdown of the independence assumption underpinning Equation (18). Independence breaks down when the coupling of allele frequency dynamics through the shared optimum displacement $\delta_{t}$ becomes important. Since disruptive selection tends to keep alleles at low frequencies where drift may overwhelm selection, many alleles experience selection only weakly, including the coupling through $\delta_{t}$.

As *L* and *μ* increase, more alleles segregate overall, while higher *N* shrinks the region where drift is important. Hence more alleles experience the $\delta_{t}$ coupling. When many alleles move together synchronously, there can be transient $V_{g}$ spikes much larger than expected if loci were independent. In Fig. 3 it can be seen that the distribution of simulated $V_{g}$ values gains a longer tail as *L*, *μ* and *N* increase, reflecting these events (see also Fig. 1). The diffusion approximation still does a reasonable job of matching the bulk of the simulated $V_{g}$ distribution, but the tails drive the mean simulated $V_{g}$ up.

We do not attempt to address this limitation of Equation (18) here since we are most interested in the lower L,μ scenarios where $V_{g}^{LB}<0.1$. For larger *L*, *μ* and *N* values our diffusion approximation for $V_{g}$ can be interpreted as an underestimate. Bulmer made some progress analyzing the joint distribution of the $p_{i}$ in the presence of the coupling induced by stabilizing selection on a quantitative trait in the simpler case of a constant optimum (Bulmer 1972).

In addition to solving Equation (18) numerically, we derive an approximate formula for $V_{g}$ (Steady-state genetic variance) that is also based on the self-consistency condition Equation (18):


(19) $$V_{g}\approx V_{g}^{LB}+\sqrt{V_{s}\sigma^{2}}$$

This approximation closely tracks the numerical diffusion approximation over much of the parameter space (solid lines in Figs. 2 and 3). Equation (19) starts to break down if selection on individual alleles becomes too weak relative to drift or the mutational influx becomes too small: formally we require, $2L\mu V_{s}\ln\;(\gamma )>\sqrt{V_{s}\sigma^{2}}$ (Steady-state genetic variance). Since Equation (19) is derived from the diffusion approximation Equation (18), it also fails to track simulations at higher *L*, *μ* and *N* (see above).

Equation (19) lets us estimate the minimum $\sigma^{2}$ required for the fluctuating optimum model to reach $h^{2}=0.1$ in a case where the LB model is failing (i.e. $V_{g}^{LB}\approx 0$). Namely $\sigma^{2}∼10^{-3}$, in agreement with our simulation results.

### Required mutational target sizes

We now address a standard critique of the LB model (and house of cards approximation), and by extension our broader understanding of quantitative genetic variation (Turelli 1984; Johnson and Barton 2005; Huang et al. 2016; Walsh and Lynch 2018).

LB model predictions are often analyzed in terms of the *L* that would be needed to reach observed heritabilities. The reason for this is that LB model predictions depend only on *μ*, $V_{s}$ and *L*, of which *L* is the least certain. *μ* can be measured fairly accurately by mutation accumulation (Huang et al. 2016). For $V_{s}$ we operate under the standard assumption that $V_{s}>20$ is unlikely for many traits of interest (Johnson and Barton 2005).

**Fig. 4.**
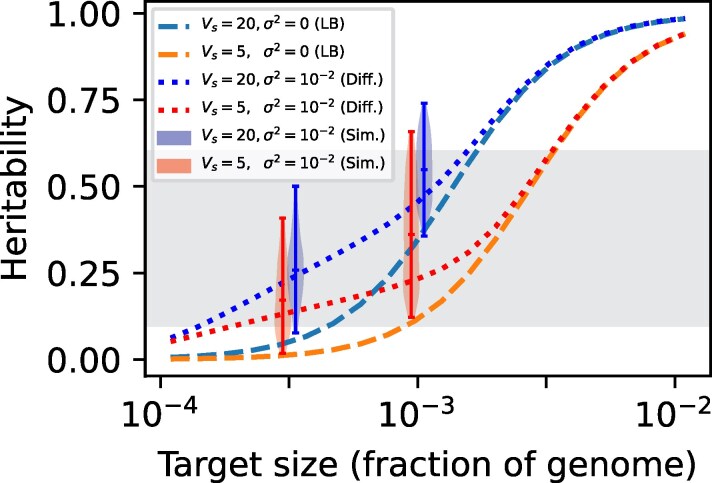
The mutational target size (proportional to *L*) needed to reach $h^{2}=0.1$ is reduced by more than an order of magnitude in the fluctuating optimum model. Violinplot shows the same simulation outputs as Fig. 2. Diffusion approximation in solid lines, LB model prediction in dashed lines. Other parameters: $2N=10^{4},a^{2}=10^{-1},\mu =6.6\times 10^{-6}$.

In Fig. 4 we show the LB and fluctuating optimum model predictions as functions of *L*. Since our *μ* is taken from *D. melanogaster* we express *L* as a proportion of the *D. melanogaster* euchromatic genome (128Mb) following (Huang et al. 2016). The *L* needed to reach $h^{2}=0.1$ in the fluctuating optimum model is reduced by more than an order of magnitude down to around 0.01%−0.1% of total euchromatin.

## Discussion

The LB model epitomizes basic intuitions about mutation-selection balance: stabilizing selection removes variation (captured by the $V_{s}$ factor in Equation (4)) while mutation replenishes it (captured by the Lμ factor in Equation (4)). In this picture, strong stabilizing selection is a challenge for MSB models (Johnson and Barton 2005; Walsh and Lynch 2018), one that might be overcome by massive mutational target sizes (Sella and Barton 2019).

The fluctuating optimum model developed here makes surprisingly different predictions given that the only change is from a stationary optimum to slow, undirected movement of the optimum. In the fluctuating optimum model, there is an additional component of $V_{g}$ given approximately by $\sqrt{V_{s}\sigma^{2}}$, which makes up the bulk of $V_{g}$ in the cases where the LB heritability is close to zero. The square-root dependence on $\sigma^{2}$ implies that even small $\sigma^{2}$ can have a surprisingly large impact. The LB model can thus be seen as a $\sigma^{2}=0$ edge case unrepresentative of $\sigma^{2}>0$ models even very close to it. Thus, the LB predictions may be a substantial underestimate if there is reason to doubt that the optimum is static, which there typically will be.

It may seem obvious from Equation (2) that even slow optimum movement can produce qualitatively different predictions. The optimum displacement |δ| only needs to be of order *a* (the effect of one allele) for the directional term $a\delta /V_{s}$ to be as important as the disruptive term $a^{2}/2V_{s}$. However, this reasoning does not account for the fact that the optimum displacement is continually changing in both magnitude and direction in the fluctuating optimum model. A fairly lengthy analysis was needed to show the conditions under which fluctuations overcome disruptive selection.

Interestingly, similar results are obtained by Waxman and Peck (1999) assuming constant optimum movement at velocity *α* instead of stochastic fluctuations. The leading order contribution to $V_{g}$ assuming $V_{g}^{LB}\ll 1$ is $V_{g}=f(m,\mu )\sqrt{2LmV_{s}\alpha }$ where $f(m,\mu )=[8\ln\;(\frac{m^{2}}{2\mu V_{s}}){]}^{-\frac{1}{4}}$ and *m* is a mutation effect size parameter analogous to *a*. This formula has square root dependence on $V_{s}$ and *α*, analogous to $\sqrt{V_{s}\sigma^{2}}$ in our approximate solution for $V_{g}$ (Equation (19)).

Our approximate expression for the variance added by fluctuations $\sqrt{V_{s}\sigma^{2}}$ is independent of *L* and *μ*. This is surprising because genetic variance in the fluctuating optimum model is maintained by a turnover of new mutations (Mechanism generating diversity). The full diffusion approximation is in fact dependent on *L* and *μ* particularly for lower mutations rates $\mu <10^{-6}$, although the dependence is much weaker than the linear dependence of the *LB* model (Fig. 3). The feedback between $V_{g}$ and selection coefficient fluctuations reduces the sensitivity of $V_{g}$ to the influx of mutations. An analogous phenomenon occurs in spatially heterogeneous populations where $V_{g}$ depends more strongly on the local spatial gradient in the optimum than the mutational input (Polechová and Barton 2015).

However, the diffusion approximation for $V_{g}$ (Equation (18)) fails to capture the full complexity of the fluctuating optimum model when many coupled loci undergo concurrent frequency shifts. This produces transient events where $V_{g}$ reaches values far above the norm. Our analysis does not account for the *L* and *μ* dependence that arises from this. However, the discrepancy only starts to matter when the mutational influx is so large that the LB model falls within the observed heritability range, and this parameter regime is not our focus here.

### Multiple traits and pleiotropy

Although the fluctuating optimum model’s predictions are better than LB, they are not sufficient to explain high heritabilities in nature. Apart from incorporating more realistic environmental assumptions, the fluctuating optimum model remains heavily simplified in other respects. A particularly big limitation is the absence of multiple traits and pleiotropy. The large mutation targets found in GWAS studies are not possible without pervasive pleiotropy (Sella and Barton 2019). Attempts to account for pleiotropy in simple heritability models have faced similar issues as the LB model. For instance, apparent selection models, which assume that stabilizing selection on individual traits results solely from pleiotropy between the trait and fitness, are unable to reproduce the observed intensity of selection and have pathological dependence on *N* (Johnson and Barton 2005).

Extending Equation (2) to M>1 traits is straightforward in the simple case where $V_{s}$ is the same for all traits. The variables *z*, $z^{*}$, $\delta_{t}$ and $a_{i}$ are simply replaced with length *M* vectors where each entry corresponds to one trait. Equations (1) and (2) still hold, with products like $(z-z^{*}{)}^{2}$ and $a_{i}\delta$ replaced with the vector dot product. However, Equation (6) no longer holds because the analog of Equation (5) is


(20) $$\frac{d{\bar{z}}_{m}}{dt}\approx \sum_{i=1}^{L}a_{mi}\sum_{m=1}^{M}a_{mi}\delta_{mt}.$$

where the subscript *m* denotes the component of $\bar{z}$, *a* and $\delta_{t}$ corresponding to trait *m*. As a result, the multi-trait generalization of Equation (6) is a coupled system of SDEs for $\delta_{mt}$ that depends on all of the $a_{mi}$. Even if we simplify by only allowing one effect size as we did in the single-trait model, this system is not expressible in terms of the $V_{g}$ for each trait, which was essential to our analytical approach.

Moreover, a multi-trait version of the fluctuating optimum model requires assumptions about the relationship between the effects of mutations on different traits. Assuming symmetric effects as done in (Simons et al. 2018) is problematic because the effect size *a*—and hence the $h^{2}$ associated with any one trait—declines with the number of traits assumed to be present for a given *s*. It also seems unlikely that mutations with larger phenotypic effects would be symmetric in this way, yet these mutations will respond most strongly to optimum fluctuations.

Given these complications we do not pursue multi-trait generalizations of the fluctuating optimum model in this manuscript, although we suggest this is a promising avenue for future work.

### Constraints on optimum movement

The Brownian model of optimum movement breaks down if the long-run variance in the trait optimum is comparable to $V_{e}$ (or smaller) in an OU model of trait optimum movement (Ornstein-Uhlenbeck process for $\delta_{t}$). In this regime, the variance and autocovariance of $\delta_{t}$ are appreciably reduced, diminishing the impact of optimum movement on $V_{g}$. This analytical prediction is confirmed with simulations assuming OU optimum movement for varying strengths of the OU restoring force parameter denoted *r* (Supplementary Figs. 1–4 in File S1).

We have assumed that we are not in this tightly constrained environmental regime. Analysis of this regime could be an interesting avenue for future work. Ideally, however, we would have better empirical estimates of *r* and also $\sigma^{2}$. Similar quantities are routinely estimated in the phylogenetic comparative literature but it is not apparent that existing estimates are applicable since these analyses span longer timescales than we analyze here and also rely on comparing the evolution of different species (Hansen 1997).

### Strength of selection and variable effect sizes

In contrast to LB where stronger selection strictly reduces $h^{2}$, to see a meaningful increase in $h^{2}$ in the fluctuating optimum model we need selection to be strong relative to drift (Ns>1). In reality many alleles presumably fall below the Ns∼1 threshold, and will be governed by a balance between mutation and drift, not selection (Bürger et al. 1989). A purely neutral scenario does not appear to be compatible with empirical findings (Huang et al. 2016; Walsh and Lynch 2018), so presumably a range of effect sizes are present potentially including a substantial fraction of variants with Ns>1. In a more realistic version of the present model that included a range of effect sizes, we expect that only larger effect size loci with Ns>1 would be affected by fluctuations.

### Future directions

In the simplified form developed here, the fluctuating optimum model is primarily instructive as an insight into the powerful effects of even slowly accumulating fluctuations in the optimum, rather than a fully-fledged explanation of observed $h^{2}$ values. What we claim our results *do* show is that simple models of quantitative genetic variation can attain observed values of $h^{2}$ without having to assume extreme mutational target sizes, contrary to long-standing consensus (Johnson and Barton 2005; Huang et al. 2016; Walsh and Lynch 2018). This finding is important because, if MSB is responsible for high trait heritabilities, we expect that even a simplified accounting of the dominant effects of mutation and selection would predict heritabilities that are at least the correct order of magnitude. Failing this we are faced with two options.

First, we could embrace the main alternative hypothesis to MSB: balancing selection (selection that preserves polymorphism). Currently there is no empirical support for balancing selection being sufficiently widespread to explain consistently high heritabilities (Sella and Barton 2019). Moreover, from a theoretical perspective, models of balancing selection almost always require large selection coefficients and organism-specific assumptions about the form of selection (e.g. particular life histories), raising the question of how balancing selection could explain consistently high heritability even in principle (Bertram and Masel 2019). On the other hand, some manifestation of mutation-selection-balance is almost certainly present to a substantial extent in every trait/species.

This leaves the second option: the failure of simple quantitative genetic models indicates fundamental holes in our understanding of quantitative trait evolution (Johnson and Barton 2005; Huang et al. 2016; Walsh and Lynch 2018). Our findings suggest that perhaps one big hole is environmental change, which has either been omitted or modeled in ways that have not captured the potential generality and magnitude of its impact.

Many aspects of our model could be made more realistic including allowing multiple traits and pleiotropy, linkage disequilibrium, and different effect sizes. We hope these will be pursued in future work. However, we emphasize that the mechanism driving increased heritability in the fluctuating optimum model is not necessarily sensitive to these factors. The essence of the variance-increasing effect captured by the fluctuating optimum model is a feedback between adaptation rate and selection coefficient fluctuations. We anticipate similar effects on heritability will occur in more complicated models provided the optimum moves in a comparable way (i.e. a undirected stochastic movement with long-term variance $>V_{e}$) with sufficiently strong selection on a sufficiently large proportion of the underlying alleles.

## Supplementary Material

iyag063_Supplementary_Data

## Figures and Tables

**Fig. A1.**
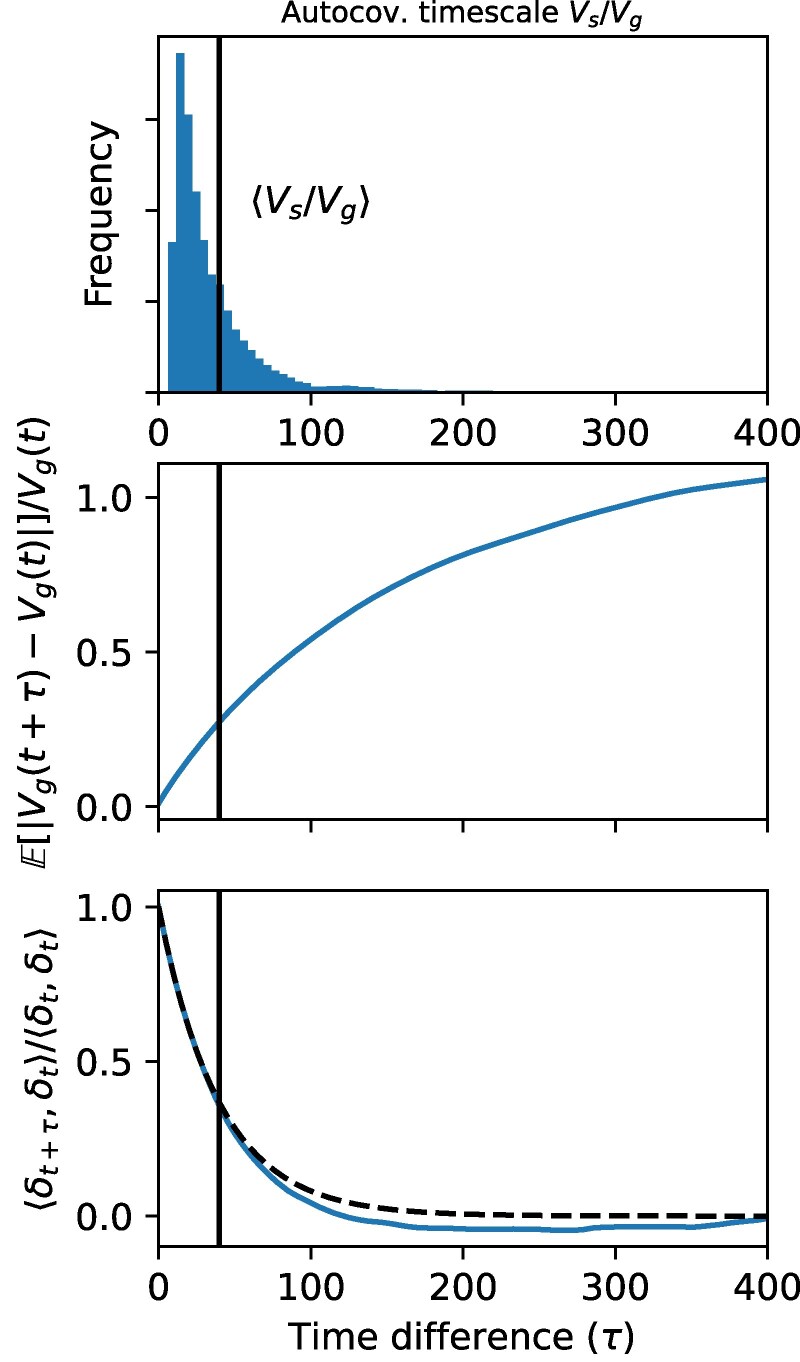
Top panel: Distribution of autocorrelation timescales $\frac{V_{s}}{V_{g}}$ over a simulation run of $10^{5}$ generations. Vertical black line: mean autocovariance timescale $\langle \frac{V_{s}}{V_{g}}\rangle \approx 40$. Middle panel: Mean absolute relative change $E[|V_{g}(t+\tau )-V_{g}(t)|]/V_{g}(t)$ in $V_{g}$ after *τ* generations. Bottom panel: Autocorrelation in *δ*. Dashed line shows the exponential theoretical formula Equation (7) using the mean autocovariance timescale. Parameters: $L=100,\sigma^{2}=10^{-2},2N=10^{4},a^{2}=0.1,\mu =6.6\times 10^{-6}$.

## Data Availability

Code and data used to generate figures can be accessed at github.com/jasonbertram/fluctuating_optimum Supplemental material available at GENETICS online.

## Appendices

### Ornstein-Uhlenbeck process for $\delta_{t}$

Focusing on the directional pull to the optimum and ignoring the effects of drift and disruptive selection (which are negligibly weak by comparison), we have $\frac{dp_{i}}{dt}=\frac{a_{i}\delta }{V_{s}}p_{i}(1-p_{i})$ and so

(A1 )
$$\frac{d\bar{z}}{dt}=2\sum_{i}a_{i}\frac{dp_{i}}{dt}=v\delta .$$

where $v=\frac{V_{g}}{V_{s}}$.

With a restoring force r>0 on the optimum, the evolution of $\bar{z}$ is described by a coupled system of SDEs

(A2 )
$$\left[\begin{array}{c} dz_{t}^{*}\\ d{\bar{z}}_{t} \end{array}\right]=-\left[\begin{array}{cc} -r & 0\\ v & -v \end{array}\right]\left[\begin{array}{c} z_{t}^{*}\\ {\bar{z}}_{t} \end{array}\right]\;dt+\sigma \left[\begin{array}{c} 1\\ 0 \end{array}\right]\;dW_{t}$$

The first row above is the OU process for $z_{t}^{*}$, which has steady-state variance $\frac{\sigma^{2}}{2r}$. On the other hand, the steady-state temporal autocovariance of $\delta_{t}$ can be computed as (Gardiner 2009, Sec. 4.5.6b,d)

(A3 )
$$\langle \delta_{t},\delta_{t-\tau }\rangle =\frac{\sigma^{2}}{2\left(v^{2}-r^{2}\right)}\left(ve^{-v\tau }-re^{-r\tau }\right).$$

where we have assumed v≠r.

Assuming that the environment is not tightly constrained in the sense that $\frac{\sigma^{2}}{2r}\gg 1$, then since $\sigma^{2}\leq 0.01$ we require $r\ll 5\times 10^{-3}$. For a typical scenario with $V_{g}=0.1$ and $V_{s}\leq 20$ we therefore have r≪v. Hence Equation (A2) reduces to Equation (7) as though the optimum was following a Brownian motion process.

### Approximate constancy of $V_{g}$

The rate at which $V_{g}$ changes is

(A4 )
$$\frac{dV_{g}}{dt}=2a^{2}\sum_{i}\frac{d}{dt}[p_{i}(1-p_{i})]=2\sum_{i}a_{i}^{2}(1-2p_{i})\frac{dp_{i}}{dt}$$

where

$$\frac{dp_{i}}{dt}\approx \frac{a_{i}}{V_{s}}\delta p_{i}(1-p_{i})$$

because allele frequency change is dominated by the directional component during a return to the optimum.

On the other hand, the rate at which the mean trait value changes is

$$\frac{\bar{z}}{dt}=2\sum_{i}a_{i}\frac{dp_{i}}{dt}$$

Comparing these rates, we see in most cases $\left|\frac{dV_{g}}{dt}|\ll |\frac{\bar{z}}{dt}\right|$. These sums differ only due to the presence of an additional factor of $a_{i}(1-2p_{i})$ in Equation (A4). By assumption $a_{i}^{2}\leq 0.1$. We also have $|1-2p_{i}|\leq 1$ and moreover, alleles at intermediate frequencies which make the largest individual contributions to $d\bar{z}/dt$ induce negligible change in $V_{g}$. Additionally, all terms in $d\bar{z}/dt$ have the same sign, whereas the terms in $dV_{g}/dt$ will often have opposite signs, partly cancelling each other out.

Therefore, over the autocovariance timescale $\tau_{a}$, which is also the minimal timescale required for appreciable change in $\bar{z}$, the change in $V_{g}$ will not be large enough to warrant the complication of treating its full time-dependence. Fig. A1 shows the magnitude of change in $V_{g}$ at different timescales in an L=100 simulation run. The average absolute relative change in $V_{g}$ at the mean autocovariance timescale is ≈25%. This amount of change in $V_{g}$ is not sufficient to meaningfully perturb the statistical properties of the $\delta_{t}$ process, which closely follows the theoretical prediction Equation (7) assuming a constant $V_{g}$.

### Averaging the autocovariance

Incorporating genetic drift into Equation (2), we obtain

(A5 )
$$\frac{\partial \phi }{\partial t}=\left[A-\frac{\partial }{\partial p}\frac{a\delta_{t}}{V_{s}}p(1-p)\right]\phi$$

where we have introduced the operator

(A6 )
$$A=-\frac{\partial }{\partial p}s(2p-1)p(1-p)+\frac{1}{4N}\frac{\partial^{2}}{\partial p^{2}}p(1-p)$$

and ϕ=ϕ(p,t|p(0)) is the probability density function for an allele to have frequency *p* at time *t* given it had frequency p(0) at t=0.

The stochastic variable $\delta_{t}$ obeys Equation (6) and has autocovariance timescale $\tau_{a}$. We now remove autocovariance from Equation (A5) using the cumulant expansion (Van Kampen 1974, 1992), which effectively integrates the behavior of $\delta_{t}$ over the interval $\tau_{a}$ and gives an approximate “effective” uncorrelated diffusion model. This approximation requires two conditions:

1. Fluctuations effectively behave like small uncorrelated pulses when viewed in time increments of $\tau_{a}$, allowing them to be described as part of an (uncorrelated) diffusion model. Formally, “small” here means that we require (Van Kampen 1992, pp. 400)
  (A7 ) $$\frac{a}{V_{s}}|\delta_{t}|\tau_{a}\ll 1,$$
  where
  $$|\delta_{t}|=\langle \delta_{t},\delta_{t}\rangle^{\frac{1}{2}}=\sigma {\left(\frac{\tau_{a}}{2}\right)}^{\frac{1}{2}},$$
  and so
  $$\frac{a}{V_{s}}|\delta_{t}|\tau_{a}=\frac{\sigma a\tau_{a}^{\frac{3}{2}}}{\sqrt{2}V_{s}}.$$
  Now choose $V_{s}=5$, $\sigma^{2}=10^{-2}$ and $a^{2}=0.1$ i.e. the worst case choices in each parameter’s assumed range for satisfying Equation (A7). This gives
  $$\frac{a}{V_{s}}|\delta_{t}|\tau_{a}=\frac{(1/V_{g}{)}^{\frac{3}{2}}}{20}$$
  Thus, Equation (A7) is satisfied if $h^{2}=\frac{1}{2}$, but starts to break down as $h^{2}$ falls to $\frac{1}{10}$. This is the worst case; for other parameter choices smaller $h^{2}$ will satisfy Equation (A7).
  In addition to Equation (A7), we require that fluctuations are uncorrelated after $\tau_{a}$ timesteps. Of course this is only an approximation because $\tau_{a}$ is an exponential decay timescale for the autocorrelations in $\delta_{t}$ (see bottom panel in Fig. A1). This approximation is conservative i.e. it underestimates the influence of fluctuations (see below for further discussion).
2. Allele frequency change due to *A* (disruptive selection + drift) must be negligible over the interval $\tau_{a}$ (Van Kampen 1992, pp. 401). This condition is not satisfied near p=0,1 where drift dominates over selection. However, in practice this boundary behavior is not important because both the exact and approximated fluctuations are negligibly small near p=0,1. What does matter is the component of *A* due to disruptive selection, which starts to exceed the change due to drift at the frequency scale $p^{*}∼\frac{1}{Ns}$(Desai and Fisher 2007). Formally, we require
  (A8 ) $$|s(1-2p^{*})|\tau_{a}\ll 1$$
  Similar to the first condition above, we assume without loss of generality that $a^{2}=0.1$, $V_{s}=5$ and 2N=1000. Then $p^{*}\ll 1$ and Equation (A8) reduces to the timescale ratio in Equation (9). Similar to Equation (A7), this condition is comfortably satisfied at $h^{2}=0.5$, but starts to break down as we approach $h^{2}=0.1$.

When the above conditions break down, the approximate averaged diffusion model underestimates the influence of fluctuations since it effectively discards autocovariance that persists between the averaging intervals of length $\tau_{a}$. Treating fluctuations as though they truncate rapidly in the low $h^{2}$ situation where they do not underestimates the $V_{g}$ increase that they cause—some positive autocovariance is effectively being omitted. But, in practice, since $h^{2}$ is already close to zero, underestimating in this case $V_{g}$ makes little difference.

Performing the cumulant expansion only retaining the first fluctuation term (Van Kampen 1992, pp. 401), and accelerating time to be in units of 2N generations, we obtain the diffusion equation

(A9 )
$$\begin{array}{rl} \frac{\partial \bar{\phi }}{\partial t} & =-\frac{\partial }{\partial p}\left[\left(\frac{\beta }{2}-\gamma \right)(1-2p)p(1-p)\bar{\phi }\right]\\ & \;+\frac{1}{2}\frac{\partial^{2}}{\partial p^{2}}\left[\left(1+\beta p(1-p)\right)p(1-p)\bar{\phi }\right] \end{array}$$

where $\bar{\phi }$ is *ϕ* averaged over the autocorrelation timescale, γ=2Ns, $\beta =2N\sigma_{a}^{2}$ and

(A10 )
$$\sigma_{a}^{2}=2\frac{a^{2}}{V_{s}^{2}}\int_{0}^{\infty }\langle \delta_{t},\delta_{t-\tau }\rangle d\tau =\frac{a^{2}}{V_{s}^{2}}\frac{\sigma^{2}}{(V_{g}/V_{s}{)}^{2}}=\frac{a^{2}\sigma^{2}}{V_{g}^{2}}$$

### Steady state density

To derive the steady state density we first incorporate mutations into the above diffusion equation by adding μ(1−2p) to the mean term. The steady state solution of Equation (A9) is then obtained by setting $\frac{\partial \bar{\phi }}{\partial t}=0$ and integrating, which gives

(A11 )
$$\frac{d}{dp}\left(V(p)\bar{\phi }\right)-M(p)\bar{\phi }=0,$$

where

(A12 )
$$M(p)=\left(\frac{\beta }{2}-\gamma \right)(1-2p)p(1-p)+(1-2p)2N\mu$$

(A13 )
$$V(p)=\frac{1}{2}[1+\beta p(1-p)]p(1-p).$$

Note that the integration constant is zero (i.e. zero probability flux) due to the symmetry of the model about $p=\frac{1}{2}$. Multiplying Equation (A11) by $Ve^{-\int \frac{M(p)}{V(p)}\;dp}$ and integrating gives Equation (13).

### Steady-state genetic variance

The distribution $\bar{\phi }$ allows us to compute the steady-state additive genetic variance from Equation (18). It is possible to evaluate this integral in terms of hypergeometric functions, although we did not find this step helpful as we were unable to find a good way to productively truncate the infinite sums they involve. Here we derive an alternative approximation that works well in the parameter regime of interest.

Under the present parameters we have θ≪1, and so $[p(1-p){]}^{\theta -1}\approx [p(1-p){]}^{-1}$. Hence the integrand is approximately

$$\begin{array}{rl} \;p(1-p)\bar{\phi }(p) & \approx C{\left[1+\beta p(1-p)\right]}^{-b}\\ & =C{\left[\beta (p+d)(1+d-p)\right]}^{-b} \end{array}$$

where b=2γ/β+θ and $d=-\frac{1}{2}+\sqrt{\frac{1}{4}+\frac{1}{\beta }}$. This function is symmetric about $p=\frac{1}{2}$, and since b>0, the points p=−d<0 and p=1+d>1 are singular. $\bar{\phi }(p)$ thus changes most rapidly near p=0,1. In particular, the factor $(p+d{)}^{-b}$ changes much faster than the factor $(1+d-p{)}^{-b}$ in the neighbourhood of p=0. We approximate the integrand by introducing an effective exponent $\tilde{b}$ that satisfies

$$d^{\tilde{b}}(p-d{)}^{-\tilde{b}}\approx [c(p+d)(1+d-p){]}^{-b}$$

near p=0. Matching the gradients of these functions gives

$$\tilde{b}=\frac{b}{d+1}.$$

We now integrate:

$$\begin{array}{rl} \int_{0}^{1}p(1-p)\bar{\phi }(p)\;dp & =2\int_{0}^{\frac{1}{2}}\;p(1-p)\bar{\phi }(p)\;dp\\ & \approx 2Cd^{\tilde{b}}\int_{0}^{\frac{1}{2}}(p+d{)}^{-\tilde{b}}\;dp\\ & =\frac{2Cd^{\tilde{b}}}{\tilde{b}-1}\left[d^{1-\tilde{b}}-{\left(\frac{1}{2}+d\right)}^{1-\tilde{b}}\right] \end{array}$$

To evaluate the normalization constant *C* we use the fact that $\bar{\phi }(p)$ is singular at p=0,1. Compared with this divergence to ∞ the change in the $[1+\beta p(1-p){]}^{-b}$ factor in $\bar{\phi }(p)$ is small. Thus we approximately evaluate *C* as

$$\begin{array}{rl} C^{-1} & \approx \int_{0}^{1}\;[p(1-p){]}^{\theta -1}\;dp\\ & \approx 2\int_{0}^{1}\;p^{\theta -1}\;dp\\ & =\frac{2}{\theta } \end{array}$$

Therefore

(A14 )
$$V_{g}=8L\mu Na^{2}\frac{d^{\tilde{b}}}{\tilde{b}-1}\left[d^{1-\tilde{b}}-{\left(\frac{1}{2}+d\right)}^{1-\tilde{b}}\right]\equiv f(V_{g}).$$

where $f(V_{g})$ is a decreasing concave-up function. The equation $V_{g}=f(V_{g})$ involves exponents of the form $V_{g}^{V_{g}^{2}}$ which makes analytical solution difficult.

Let $V_{g}^{*}$ denote the solution of Equation (A14) i.e. the intersection of $f(V_{g})$ with the one-one line. Evaluating the extremes of $f(V_{g})$ assuming $0<\sigma^{2}\ll 1$, we have

(A15 )
$$\lim_{V_{g}\to 0}f(V_{g})=4L\mu Na^{2}=8L\mu V_{s}Ns=V_{g}^{LB}2Ns$$

and

(A16 )
$$\lim_{V_{g}\to \infty }f(V_{g})=4L\mu V_{s}=V_{g}^{LB}.$$

We restrict our attention to the case where 2Ns≫1, and so

(A17 )
$$V_{g}^{LB}<V_{g}^{*}<V_{g}^{LB}2Ns$$

where the upper bound in Equation (A17) is much greater than $V_{g}^{LB}$. Therefore, depending on the shape of $f(V_{g})$, $V_{g}^{*}$ can also be much greater than $V_{g}^{LB}$.

We now derive an approximate expression for $V_{g}^{*}$. We first evaluate $f(V_{g})$ at a trial value $V_{g}=\sqrt{V_{s}\sigma^{2}}$ (this choice will be explained below). We have

$$\begin{array}{rl} \beta & =\frac{2Na^{2}\sigma^{2}}{V_{g}^{2}}=\frac{2Na^{2}}{V_{s}}=2\gamma \gg 1\\ d & \approx \frac{1}{\beta }\ll 1\\ \tilde{b} & \approx 1 \end{array}$$

and so

$$f(V_{g})\approx 2L\mu V_{s}\ln\;(2Ns)$$

It follows that

$$V_{g}^{*}\geq \min\left\{\sqrt{V_{s}\sigma^{2}},2L\mu V_{s}\ln\;(2Ns)\right\}$$

Assuming selection is sufficiently strong relative to drift (ln(2Ns)≫1) such that $2L\mu V_{s}\ln\;(2Ns)>\sqrt{V_{s}\sigma^{2}}$, we therefore have

(A18 )
$$V_{g}^{*}>\sqrt{V_{s}\sigma^{2}}$$

This assumption breaks down if *L*, *μ*, *N* or *a* is too small (see Fig. 3).

When $V_{g}^{*}=\sqrt{V_{s}\sigma^{2}}$, then we have b=1+θ≈1, which coincides with the condition Equation (15) for when fluctuations become strong enough to change the shape of $\bar{\phi }$ relative to the $\sigma^{2}=0$ case. Although Equation (A18) is only a lower bound on $V_{g}^{*}$, $V_{g}^{*}$ also cannot be much larger than $\sqrt{V_{s}\sigma^{2}}$ if $V_{g}^{LB}\approx 0$. In this case $f(V_{g})$ decreases rapidly to 0 due to the $d^{\tilde{b}}$ factor which has $V_{g}^{2}$ in the exponent, and so the intersection of $f(V_{g})$ with the one-one line occurs near $\sqrt{V_{s}\sigma^{2}}$.

In the more general situation where $V_{g}^{LB}>0$, it is harder to directly estimate the solution of $V_{g}^{*}=f(V_{g}^{*})$. Fortunately we are most interested the regime where $V_{g}^{LB}\ll 1$, which can be approximated as a small perturbation of the preceding analysis. This yields the approximation Equation (19).

### Balance of forces on allele frequencies

Here we analyze the influence of disruptive and fluctuating selection on allele frequency dynamics. We begin with the single-locus model by Karlin and Levikson (Karlin and Levikson 1974) before addressing the fluctuating optimum model.

In the Karlin-Levikson model, two alleles have fitness $1+\alpha_{1}$ and $1+\alpha_{2}$ where $\alpha_{1},\alpha_{2}$ are independent and identically distributed (iid) random variables with variance $\sigma_{KL}^{2}/2$. Therefore,

(A19 )
$$E[(\alpha_{1}-\alpha_{2}{)}^{2}]=\sigma_{KL}^{2}.$$

We relax the iid assumption to allow $\alpha_{1}$ and $\alpha_{2}$ to have different means so that new mutations are expected to be deleterious. Mathematically,

(A20 )
$$E(\alpha_{1})=-\frac{s}{2},\;E(\alpha_{2})=\frac{s}{2}$$

where *s* is a purifying selection coefficient analogous to the disruptive selection coefficient in the fluctuating optimum model.

Huerta-Sanchez et al. (2008) derived the diffusion limit for this model, which reduces to

(A21 )
$$\begin{array}{rl} \frac{\partial \phi (p,t)}{\partial t} & =-\frac{\partial }{\partial p}\left[\left(-s+\frac{\sigma^{2}}{2}(1-2p)\right)p(1-p)\phi (p)\right]\\ & \;+\frac{1}{2}\frac{\partial^{2}}{\partial p^{2}}[\sigma_{KL}^{2}p^{2}(1-p{)}^{2}\phi (p)] \end{array}$$

where we omit drift from the analysis. The SDE corresponding to Equation (A21) is

(A22 )
$$\begin{array}{rl} dp(t) & =\left(-s-\frac{\sigma_{KL}^{2}}{2}(2p-1)\right)p(1-p)\;dt\\ & \;+\sqrt{\sigma_{KL}^{2}{\left(\;p(1-p)\right)}^{2}}\;dW(t) \end{array}$$

Considering first the mean term in Equation (A22), and integrating over a time interval Δt that is short enough to prevent big changes in *p*, *p* is approximately constant with respect to *t* and we have a total allele frequency change of

(A23 )
$$\begin{array}{rl} \Delta_{b}p & =\int_{0}^{\Delta t}(-s-\frac{\sigma_{KL}^{2}}{2}(2p-1))(p(1-p)\;dt\\ & \approx \left(-s-\frac{\sigma_{KL}^{2}}{2}(2p-1)\right)p(1-p)\Delta t \end{array}$$

Therefore, the relative change in frequency due to the negative shift is:

(A24 )
$$\frac{\Delta_{b}p}{p}=\left(-s-\frac{\sigma_{KL}^{2}}{2}(2p-1)\right)(1-p)\Delta t$$

Denoting the absolute relative change in frequency by *η*,

(A25 )
$$\frac{\left|\Delta_{b}p\right|}{p}=\eta$$

and solving for Δt, we have

(A26 )
$$\Delta t=\frac{\eta }{\left|-s-\frac{\sigma_{KL}^{2}}{2}(2p-1)|(1-p\right)}.$$

Next, we assess how much allele frequency change is caused by selective fluctuation during this time period. Given that we need to consider the stochastic component of Equation (A22), we use the Itô isometry

(A27 )
$$E\left[{\left(\int_{a}^{b}f(t)\;dW(t)\right)}^{2}\right]=\int_{a}^{b}E\left[\;f^{2}(t)\right]\;dt$$

where f(t) is a random function. Applying Equation (A27) to the stochastic component of Equation (A22), we obtain

(A28 )
$$\begin{array}{rl} \Delta_{a}p & =E{\left[{\left(\int_{0}^{\Delta t}\sqrt{\sigma_{KL}^{2}p^{2}(1-p{)}^{2}}\;dW(t)\right)}^{2}\right]}^{\frac{1}{2}}\\ & ={\left[\left(\int_{0}^{\Delta t}E\left(\sigma_{KL}^{2}p^{2}(1-p{)}^{2}\right)\;dt\right)\right]}^{\frac{1}{2}} \end{array}$$

Assuming again that Δt is small enough that *p* can be approximated as constant, we obtain

(A29 )
$$\Delta_{a}p\approx {\left[\sigma_{KL}^{2}p^{2}(1-p{)}^{2}\Delta t\right]}^{\frac{1}{2}}$$

and hence

(A30 )
$$\frac{\Delta_{a}p}{p}=\sqrt{\frac{\sigma_{KL}^{2}\eta (1-p)}{\left|-s-\frac{\sigma_{KL}^{2}}{2}(2p-1)\right|}}$$

Focusing on low frequencies p≪1 and setting η=1 so that Δp is of order *p*, and further requiring $\frac{\left|\Delta_{b}p\right|}{p}=\frac{\Delta_{a}p}{p}$, we finally obtain

(A31 )
$$s=\frac{3\sigma_{KL}^{2}}{2}$$

Thus, when $3\sigma_{KL}^{2}/2<s$, purifying selection drives larger changes in allele frequency than fluctuating selection, and *vice versa* when $3\sigma_{KL}^{2}/2>s$.

We now apply the same procedure to the the diffusion model in the main text, given by Equation (A9). The corresponding SDE is now:

(A32 )
$$\begin{array}{rl} dp(t) & =\left(s-\frac{\sigma_{a}^{2}}{2}\right)(2p-1)p(1-p)\;dt\\ & \;+\sqrt{\sigma_{a}^{2}{\left(\;p(1-p)\right)}^{2}}\;dW(t) \end{array}$$

where $s=\frac{a^{2}}{2V_{s}}$ now denotes the disruptive selection coefficient.

The change in frequency due to directional part of the equation is similarly

(A33 )
$$\begin{array}{rl} \Delta_{b}p & =\int_{0}^{\Delta t}\left(s-\frac{\sigma_{a}^{2}}{2}\right)(2p-1)p(1-p)\;dt\\ & \approx \left(s-\frac{\sigma_{a}^{2}}{2}\right)(2p-1)p(1-p)\Delta t. \end{array}$$

and so

(A34 )
$$\Delta t=\frac{\eta }{\left|\left(s-\frac{\sigma_{a}^{2}}{2}\right)(2p-1)(1-p)\right|}$$

where $\eta =\Delta_{b}p/p$.

Next, we apply the Itô isometry to the fluctuating selection component which leads to

(A35 )
$$\Delta_{a}p={\left[\sigma_{a}^{2}(p(1-p){)}^{2}\Delta t\right]}^{\frac{1}{2}}$$

similarly to Equation (A29).

Again setting $\left|\Delta_{a}p/p|=|\Delta_{b}p/p\right|$ and η=1, and focusing on low frequencies, we obtain a similar threshold to the single locus case

(A36 )
$$s=\frac{3\sigma_{a}^{2}}{2}$$
