# Supplementary material for: Strong amplification of quantitative genetic variation under a balance between mutation and fluctuating stabilizing selection
Source: Genetics. 2026 Mar 6;233(1):iyag063. doi: 10.1093/genetics/iyag063 (PMC13147527; doi:10.1093/genetics/iyag063)
Supplement: iyag063_Supplementary_Data [file iyag063_supplementary_data.pdf]

Supplement

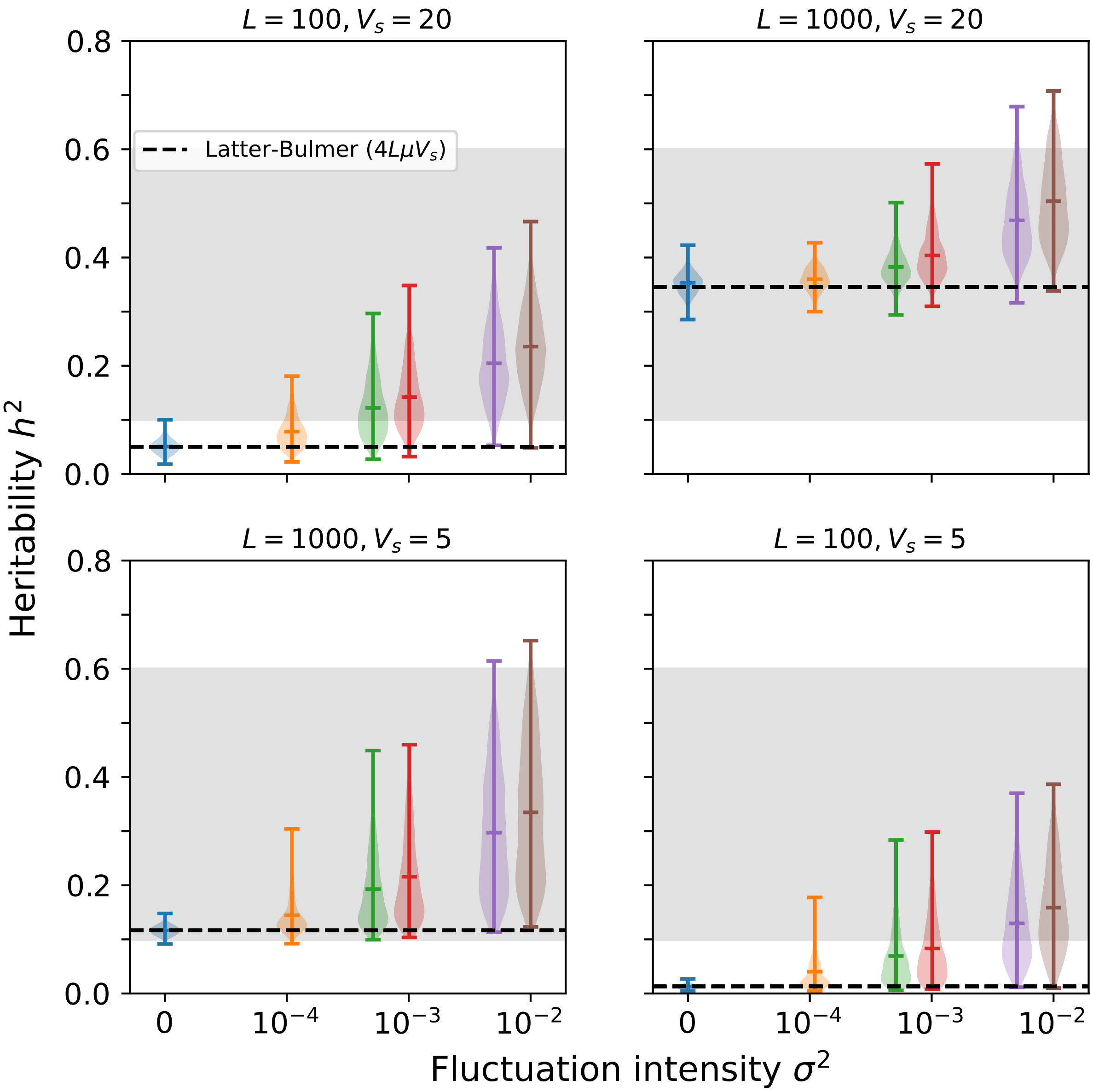

Figure 1: Same as Fig. 2 in the main text but with environmental restoring force $r = 10^{-3}$.

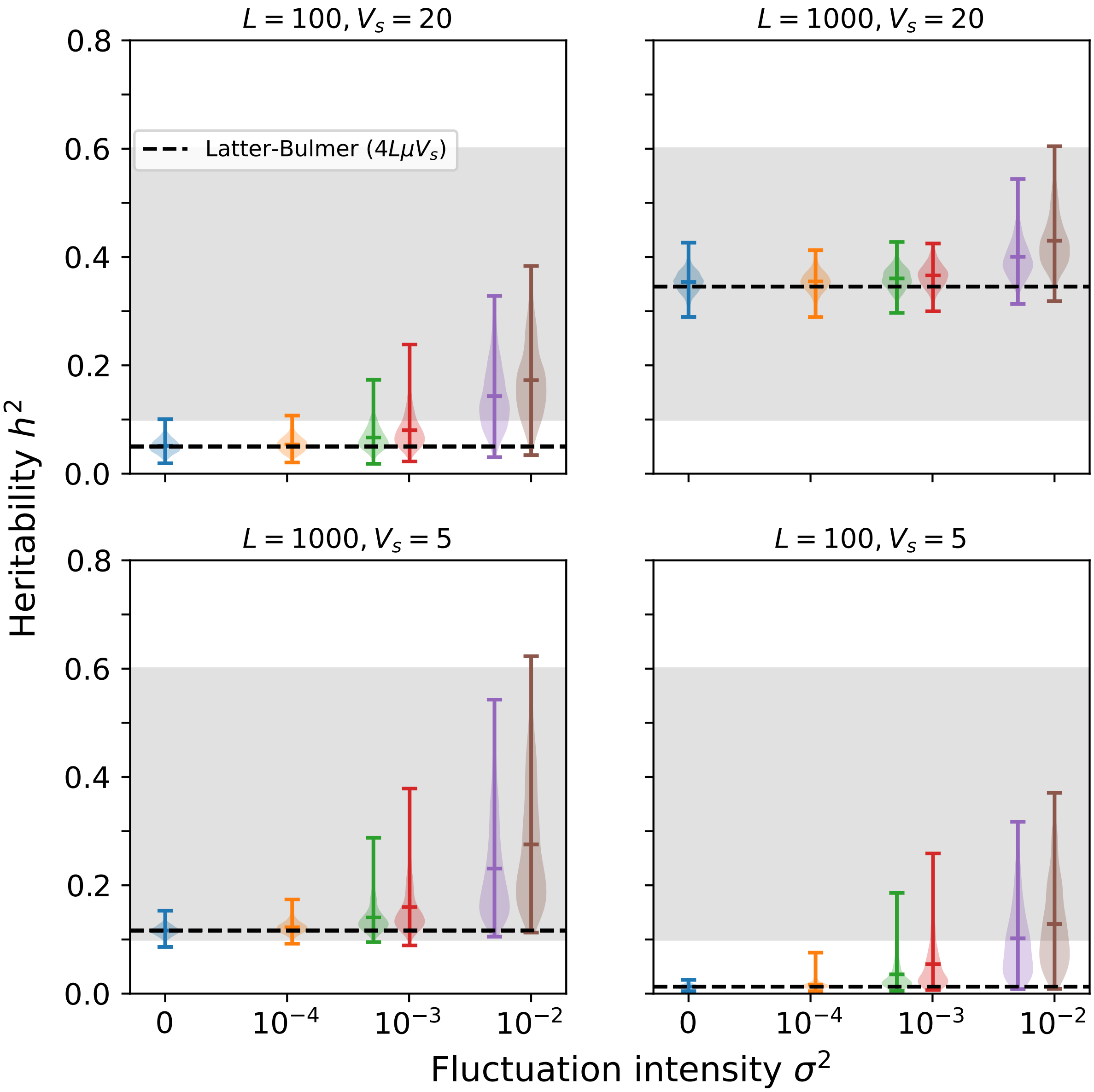

Figure 2: Same as Fig. 2 in the main text but with environmental restoring force $r = 5 \times 10^{-3}$. This case corresponds to the steady-state trait variance $\frac{\sigma^2}{2r} = 1 = V_e$.

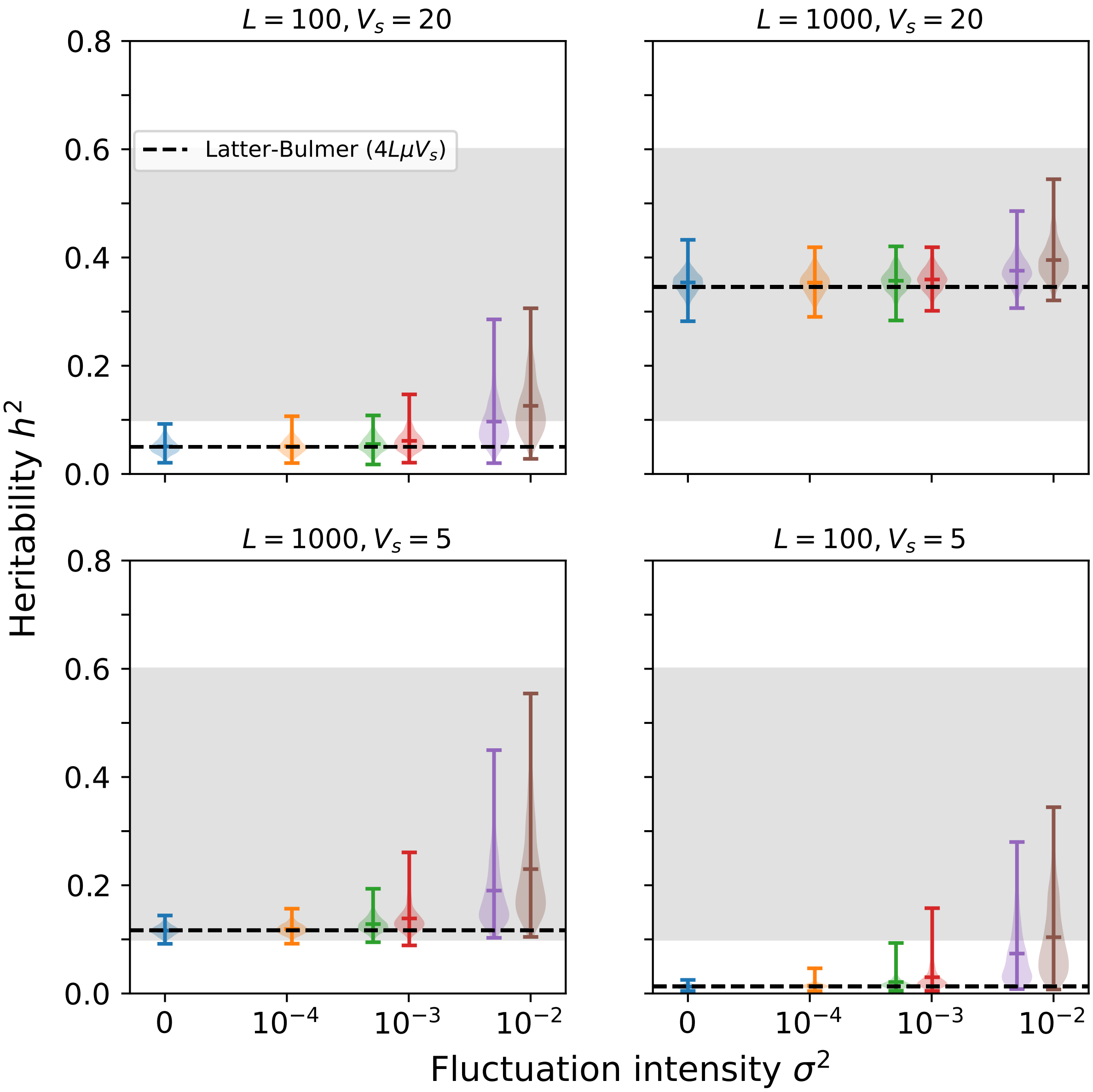

Figure 3: Same as Fig. 2 in the main text but with environmental restoring force $r = 10^{-2}$.

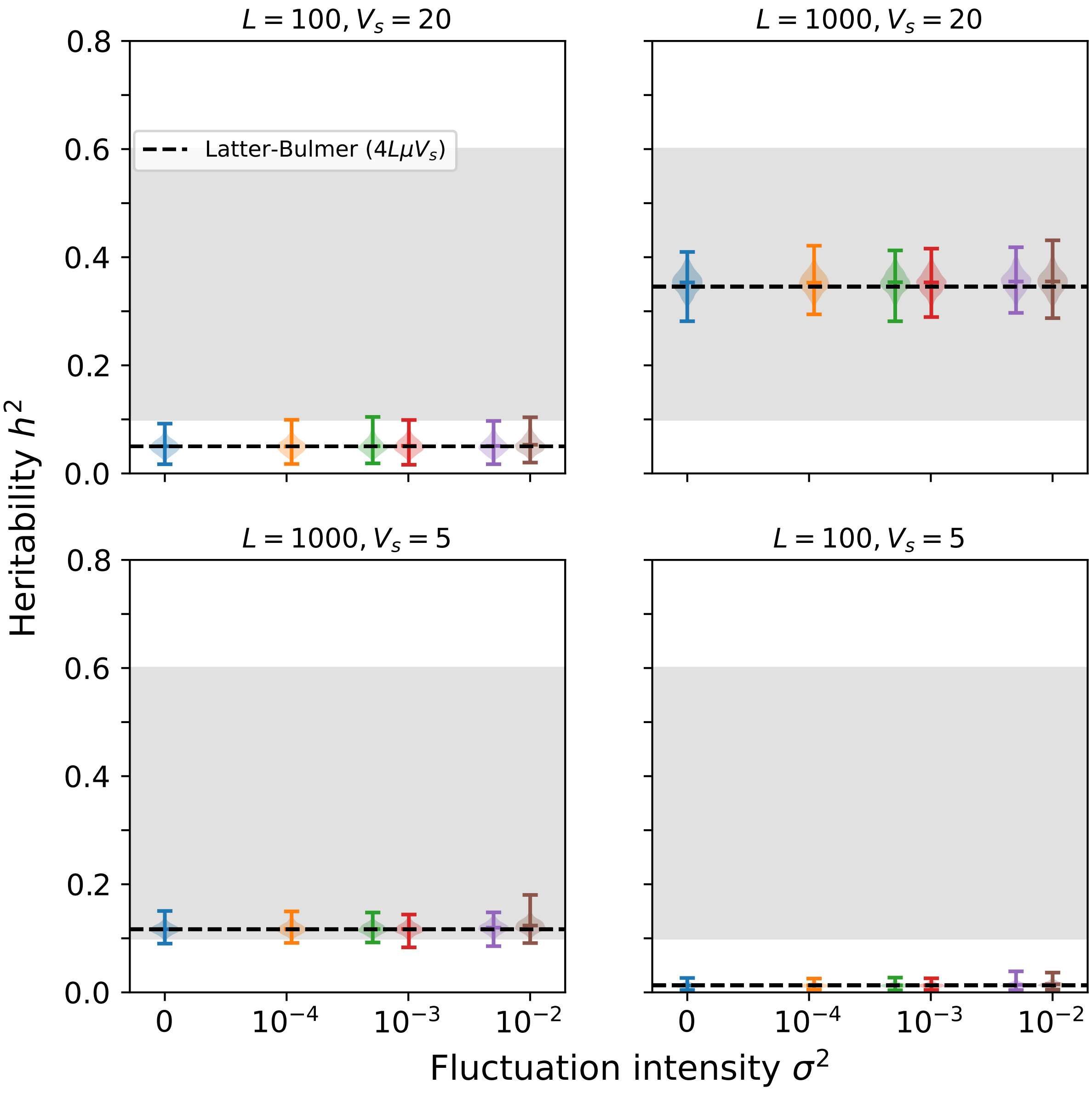

Figure 4: Same as Fig. 2 in the main text but with environmental restoring force $r = 10^{-1}$.
